# Identification of Prognostic Biomarkers Originating From the Tumor Stroma of Betel Quid-Associated Oral Cancer Tissues

**DOI:** 10.3389/fonc.2021.769665

**Published:** 2021-11-16

**Authors:** Yi-Hong Liu, Yu-Lian Chen, Ting-Yu Lai, Ying-Chieh Ko, Yu-Fu Chou, Peir-Rong Chen, Jenn-Ren Hsiao, Jang-Yang Chang, Shine-Gwo Shiah, Jeng-Woei Lee, Jia-Ling Yang, Su-Fang Lin

**Affiliations:** ^1^ Institute of Biotechnology, National Tsing Hua University, Hsinchu, Taiwan; ^2^ National Institute of Cancer Research, National Health Research Institutes, Miaoli County, Taiwan; ^3^ Institute of Bioinformatics and Structural Biology, National Tsing Hua University, Hsinchu, Taiwan; ^4^ Department of Otolaryngology, Tzu Chi University Hospital, Hualien, Taiwan; ^5^ Department of Otolaryngology, Head and Neck Collaborative Oncology Group, National Cheng Kung University Hospital, College of Medicine, National Cheng Kung University, Tainan, Taiwan; ^6^ Institute of Biotechnology and Pharmaceutical Research, National Health Research Institutes, Miaoli County, Taiwan; ^7^ Department of Life Sciences, Tzu Chi University, Hualien, Taiwan

**Keywords:** prognostic biomarkers, oral cancer, partial epithelial-mesenchymal transition (p-EMT), tumor stroma, myofibroblastic CAF (myCAF), inflammatory CAF (iCAF), hyaluronidase

## Abstract

**Background:**

Partial epithelial-mesenchymal transition (p-EMT) is a distinct clinicopathological feature prevalent in oral cavity tumors of The Cancer Genome Atlas. Located at the invasion front, p-EMT cells require additional support from the tumor stroma for collective cell migration, including track clearing, extracellular matrix remodeling and immune evasion. The pathological roles of otherwise nonmalignant cancer-associated fibroblasts (CAFs) in cancer progression are emerging.

**Methods:**

Gene set enrichment analysis was used to reveal differentially enriched genes and molecular pathways in OC3 and TW2.6 xenograft tissues, representing mesenchymal and p-EMT tumors, respectively. R packages of genomic data science were executed for statistical evaluations and data visualization. Immunohistochemistry and Alcian blue staining were conducted to validate the bioinformatic results. Univariate and multivariate Cox proportional hazards models were performed to identify covariates significantly associated with overall survival in clinical datasets. Kaplan–Meier curves of estimated overall survival were compared for statistical difference using the log-rank test.

**Results:**

Compared to mesenchymal OC3 cells, tumor stroma derived from p-EMT TW2.6 cells was significantly enriched in microvessel density, tumor-excluded macrophages, inflammatory CAFs, and extracellular hyaluronan deposition. By translating these results to clinical transcriptomic datasets of oral cancer specimens, including the Puram single-cell RNA-seq cohort comprising ~6000 cells, we identified the expression of stromal *TGFBI* and *HYAL1* as independent poor and protective biomarkers, respectively, for 40 Taiwanese oral cancer tissues that were all derived from betel quid users. In The Cancer Genome Atlas, *TGFBI* was a poor marker not only for head and neck cancer but also for additional six cancer types and *HYAL1* was a good indicator for four tumor cohorts, suggesting common stromal effects existing in different cancer types.

**Conclusions:**

As the tumor stroma coevolves with cancer progression, the cellular origins of molecular markers identified from conventional whole tissue mRNA-based analyses should be cautiously interpreted. By incorporating disease-matched xenograft tissue and single-cell RNA-seq results, we suggested that *TGFBI* and *HYAL1*, primarily expressed by stromal CAFs and endothelial cells, respectively, could serve as robust prognostic biomarkers for oral cancer control.

## Introduction

In Taiwan, the population of betel quid users significantly declined *via* a successful nationwide oral cancer screening program initiated 22 years ago. However, the 5-year survival rate (~ 56%), death ranking in all cancers (fourth for males, sixth for all), and death of middle age (60 as male median) have remained serious concerns in recent years (1). In addition, a retrospective study indicated that approximately one-third of oral cancer patients had local recurrence (34.6%, 146/422), and approximately one-fifth of 5-year survivors still experienced recurrence (18.1%, 23/127) (2). These data prompted local researchers to devote more efforts to encouraging hesitant patients for curative surgery (3), setting optimal measures for adjuvant radiotherapy (50–60 Gy) (4) and adequate surgical margins (≥ 5 mm for good overall survival) (5), among others. Regrettably, molecular biomarkers that can reliably forecast oral cancer prognosis are still unavailable.

In tumor biology, partial epithelial-mesenchymal transition (p-EMT), a.k.a. hybrid E/M status, EMT continuum or EMT spectrum, is referred to as varied intermediate stages where epithelial cells dedifferentiate to their mesenchymal counterparts (6). In contrast to fully mesenchymal cancer cells that invade alone, p-EMT cells migrate collectively and directionally in the tumor stroma, notably angiolymphatic and perineural invasions. During cluster advancement, intercellular adherent junctions and cadherins are responsible for multicellular integrity and cell-cell coordination; extracellular matrix metalloproteinases (MMPs) and basement membrane type IV collagens are essential for track clearing and secondary extracellular matrix remodeling, respectively (7).

Previously, Puram et al. revealed the ecosystems of ~6,000 cells from 18 treatment-naïve oral cancer specimens, including 5 matched lymph node metastases, by using high-resolution single-cell RNA sequencing (scRNA-seq). Their results indicated that greater than 70% of oral cavity tumors in the cancer genome atlas (TCGA) are malignant-basal type, which displays either EMT or p-EMT as hallmarks (8). Subsequent experiments using quantitative immunohistochemistry assays (PDPN, LAMB3, LAMC2) further revealed that p-EMT is statistically associated with nodal metastasis and perineural invasion in 99 primary oral cancer tissues, providing p-EMT as a useful indicator for decision-making intraoperatively (e.g., N0 neck dissection) or postoperatively (e.g., adjuvant therapy) (9).

Independently, our prior study showed that DDR1, COL4A5, COL4A6 and PDPN are statistically associated with angiolymphatic invasion in matched tumor-adjacent normal tissues from 40 Taiwanese oral cancer patients. In addition, inhibition of DDR1 kinase activity in p-EMT oral cancer cells (TW2.6) disrupted cell cohesiveness in a 2D culture, reduced spheroid invasion in a collagen gel matrix, and suppressed angiolymphatic invasion in xenograft tissues (10). It is worth noting that compared to a mesenchymal subtype (OC3) that has a similar growth rate and clonogenicity *in vitro*, p-EMT TW2.6 repeatedly grew faster (e.g., 32 *vs*. 81 days to reach 500 mm^3^) in immunodeficient mice (NOG) despite their tumor-bearing rates were similar (11).

The tumor stroma is regarded as the nonmalignant part of a tumor that is interconnected by extracellular matrices (ECMs) with infiltrated immune cells, vascular or lymphatic vessels, and cancer-associated fibroblasts (CAFs). In head and neck cancer, tumor-stroma interactions, including tumor budding and tumor-stroma ratio, have emerged as powerful clinicopathological predictors for tumor aggression and patient survival (12–14). CAFs gained their name through the finding that activated fibroblasts proliferate and accelerate the growth of several epithelial tumors during malignant progression, a phenomenon reminiscent of wound repair and fibrosis (15, 16). Importantly, the Sahai Lab demonstrated that stromal fibroblasts are required for guiding collective cancer cell invasion of squamous cell carcinoma in an organotypic culture model (17). In addition, scRNA-seq methodology has explicitly revealed the presence of two functionally discrete CAF subtypes in clinical samples, designated myofibroblastic (myCAF) and inflammatory (iCAF) (18–22). Of importance, both variants coevolve with tumor progression in which iCAFs seemed to precede myCAFs (21, 23).

In conventional bulk transcriptomic analysis, tumor- and stroma-derived transcripts are admixed, which greatly limits precise molecular stratification and cell type-driven therapies (8, 24, 25). To circumvent this inherent ambiguity, various computational pipelines were developed to infer tumor-infiltrated stromal components in a given tissue, including ESTIMATE (26), CIBERSORTx (27), and TIMER (28). Alternatively, an RNA-seq-based hypothesis-free workflow, namely, to extract the human and the mouse reads from patient-derived xenograft tissues, followed by composite transcriptomic analysis of tumor (human) and stroma (mouse) interactions, has been established recently (29–31).

Along the same vein, our prior results showed that the stroma of mesenchymal OC3 tumors harbored a statistically higher extent of mouse fibroblasts than p-EMT TW2.6 tumors. By translating the most significantly expressed gene matrix into clinical datasets of oral cancer tissues, we showed that the summed expression of *FN1*, *TGFB2*, *TGFBR2*, and *TGFBI*, dubbed the CAF index, is a poor indicator of overall survival for oral cancer (n=40) and the PANCAN (n=9,356) cohorts (11). Here, we continued to investigate the molecular interactions between tumor cells and stromal components in p-EMT TW2.6 tumors.

## Materials and Methods

### Cell Culture, Animal Experiment, and mRNA-Seq Analysis of Xenograft Tissues

Please refer to prior study for details (11). Briefly, CGHNC8, C9, K2, K6 (32), OC3 (33), OEC-M1 (34), and TW2.6 (35) were kindly provided by researchers at distinct institutions in Taiwan. OC3 and TW2.6 were selected for two independent animal studies. We measured the tumor size and mouse weight twice a week. For Exp1, all tumors were collected on day 68. For Exp2, to obtain tumors with ~500 mm^3^ in size, tumors of TW2.6-NOG and OC3-NOG were collected on day 32 and day 81, respectively. Total RNAs of each cell line and xenograft tissue were extracted by TRIzol^®^ reagent (Invitrogen Life Technologies, Carlsbad, CA, USA), cleaned up by RNeasy column (Qiagen, Hilden, Germany), and subjected to an Agilent Bioanalyzer (Agilent, Santa Clara, CA, USA) for RNA Integrity Number (RIN) assessment. Only samples that had an RIN > 7 were selected for mRNA amplification and sequencing (stranded paired-end, Illumina platform, San Diego, CA, USA).

### Antibodies

Anti-Pecam1 (ab28364) was purchased from Abcam (Cambridge, UK); anti-Adgre1 (#70076S) was from Cell Signaling (Danvers, MA, USA).

### Immunohistochemical (IHC) Staining

Sections were dewaxed, rehydrated, and incubated with Trilogy™ (Cell Marque, Rocklin, CA, USA) (10 mM citrate buffer, pH 6.0 for Adgre1) at 121°C for 10 min to unmask antigens. At room temperature (RT), the slides were immersed in 3% hydrogen peroxide for 15 min to quench endogenous peroxidase activity followed by 1% bovine serum albumin for 60 min to block nonspecific antigenic sites. Slides were incubated with indicated primary antibodies at 4°C overnight (Pecam1, 1:100, Adgre1, 1:1500). After washing with 1X TBS containing 0.05% Tween 20, slides were incubated with horseradish peroxidase-conjugated secondary antibodies and developed by chromogen diaminobenzidine using the DakoReal™ EnVision™ kit (#K5007, DAKO, Glostrup, Denmark). All slides were counterstained with Mayer’s hematoxylin and scanned by a Pannoramic MIDI scanner (3DHISTECH, Budapest, Hungary).

### Alcian Blue Staining

Alcian blue staining kit (ab150662, Abcam, Cambridge, UK) was used to detect hyaluronan deposition in the xenograft tissues according to the manufacturer’s instruction. Briefly, dewaxed and rehydrated FFPE tissue sections were incubated with Alcian Blue Solution for 30 min at RT, slides were counterstained with Nuclear Fast Red Solution for 5 min at RT and scanned by a Pannoramic MIDI scanner (3DHISTECH).

### Quantitation of FFPE Scanned Images

To quantitate the immunostaining of Pecam1, Adgre1, and Alcian blue staining in the xenograft tissues, scanned images at 100× magnification were digitized and quantitated by using ImageJ plugged-in with the *Immunohistochemistry Image Analysis Toolbox* (v1.40p) (NIH, Bethesda, MD, USA). Quantitation results were visualized by R package *ggplot2* (v3.3.3). Statistical differences between OC3 and TW2.6 groups were evaluated by two-sample t-test of means (*compare_means*), as denoted in each plot.

### Bioinformatic Analyses

Cell line dataset (GSE150469): human reference genome (hg19) aligned reads for individual genes (n=21,916) were used to compute expression values in transcripts per kilobase million (TPM) by using *Cufflinks* (v2.1.1). R *pheatmap* (v1.0.12) was used to visualize the relative expression levels of indicated genes in each cell line. Xenograft tissue dataset (GSE149496): as described previously (11), R *XenofilteR* (v1.8) processed human (hg38) and mouse (mm10) aligned reads for individual genes were used to perform gene quantification in TPM values. The resulting gene numbers for human and mouse are 17,759 and 16,374, respectively. In the exploratory analysis, R package *limma* (v3.40.6) was used to compute differentially expressed genes followed by *Volcano* plots for visualization. To identify biological pathways enriched in the OC3- and TW2.6 tumor stroma, the mouse expression matrix was subjected to gene set enrichment analysis (GSEA) (36). Enrichment was considered significant when false discovery rate (FDR) was less than 5%. R *ggcorrplot* (v0.1.3) was used to calculate and visualize the correlation matrix comprising genes of interest. The *Pearson* correlation coefficient was computed; *p*-value < 0.05 was considered significant. R *ggplot2* (v3.3.3) was used to visualize the relative expression levels of indicated genes in the OC3- and TW2.6 stroma. Unpaired two-sample t-test of means was applied to evaluate statistical differences; *p*-value < 0.05 was considered significant. R *pheatmap* (v1.0.12) was used to visualize the relative expression levels of indicated genes in each tissue. *In silico* enumeration of cell fractions was conducted by using CIBERSORTx according to its online documentation of which the ‘single cell RNA-seq HNSCC’ and ‘LM 22’ were used as signature matrices, respectively (27). Puram scRNA-seq dataset (GSE103322): the raw expression matrix was acquired from the UCSC Cell Browser portal. Expression values (TP100K) were normalized, scaled, and log-transformed by using R *Seurat* (v4.0.3) (37). *DotPlot* was used to visualize the average expression and fraction of indicated genes across eleven cell types comprising 5,902 cells. NCKU-OrCA-40TN dataset (GSE37991): this normalized microarray dataset comprises 18,047 genes for further analysis. R *ggcorrplot* was used to calculate and visualize the correlation matrix composed of 20 selected stromal genes. The *Pearson* correlation coefficient was computed; *p*-value < 0.05 was considered significant. R *survival* (v3.2-7) was used to assess the univariate and multivariate Cox proportional hazards of indicated clinical features and genes. The Cancer Genome Atlas (TCGA) datasets: for each indicated cancer type, the expression matrix of 20 stromal genes and associated clinical information were acquired from the UCSC Xena platform (38), followed by univariate Cox proportional hazards assessment and dichotomized Kaplan–Meier overall survival curves prediction by using R *survival* (v3.2-7).

## Results

### Higher Microvessel Density Was Detected in the Mouse Stroma of the p-EMT TW2.6 Tumors

**Figure 1:**
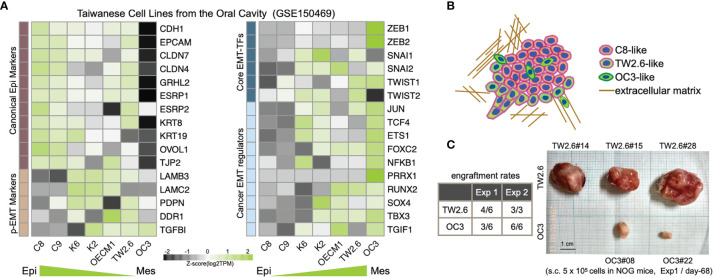
Molecular and tumorigenic features of the mesenchymal OC3 and p-EMT TW2.6 oral cancer cell lines. **(A)** Transcriptomic heatmaps of epithelial-mesenchymal transition (EMT)-related genes in five oral cancer cell lines (C8, C9, TW2.6, OECM1, OC3) and two HPV 16-E6/E7 immortalized oral keratinocyte lines (K2, K6). Epi, epithelial; Mes, mesenchymal; p-EMT, partial EMT. **(B)** A hypothetical drawing of an *in vivo* tumor tissue admixed with C8-, TW2.6- and OC3-like cells. **(C)** Engraftment rates derived from the first experiment (Exp 1) were determined at Day 68 after cell injection. In the second experiment (Exp 2), to reach a tumor mass of similar size (~ 500 mm^3^), Day 32 and Day 81 were used as sacrifice times for TW2.6 and OC3, respectively (11).

A recent consensus statement of epithelial-mesenchymal transition (EMT) research reiterated the importance of associating cellular characteristics, rather than the expression of a single or a small set of molecular markers, with EMT phenotypes (6). To comply with these guidelines, we inspected the expression levels of core EMT transcription factors, p-EMT hallmarks, and canonical epithelial markers and mesenchymal regulators (6, 9, 10, 39, 40) in a set of seven Taiwanese cell lines derived from the oral cavity. Among these, while the oral cancer cell line OC3 has the highest mesenchymal propensity, TW2.6 and OEC-M1 are hypothetically p-EMT cells since they maintain both epithelial and mesenchymal genes (**Figures 1A, B**). Indeed, we demonstrated that TW2.6, but not OC3 or OEC-M1, displayed p-EMT multicellular characteristics *in vitro* and *in vivo* (10). In addition, mesenchymal OC3 repeatedly grew slower and smaller than its p-EMT TW2.6 counterpart *in vivo* [**Figure 1C** and ref (11)], a phenomenon consistent with one prior study in that head and neck cancer tissues of the inflammatory mesenchymal subtype had a better prognosis (24).

Next, exploratory analysis of differentially expressed genes (DEGs) in the OC3 and TW2.6 xenograft tissues revealed that (1) compared to the OC3 cells expressing various innate immunity responsive genes, TW2.6 cells expressed MYC and E2F targets involved in cell proliferation and cell adhesion (**Supplementary Figure 1A**); (2) compared to the OC3 stroma harboring various extracellular matrix (ECM)- and TGFβ axis-related transcripts, the TW2.6 stroma was characterized by proangiogenic factors and immune-related genes. Gene set enrichment analysis (GSEA) also confirmed that while the epithelial-mesenchymal transition hallmark is uniquely enriched in the OC3 stroma, angiogenesis and immune-related molecular processes are recurrently detected in the TW2.6 stromal compartment (**Supplementary Figure 1B**).

**Figure 2:**
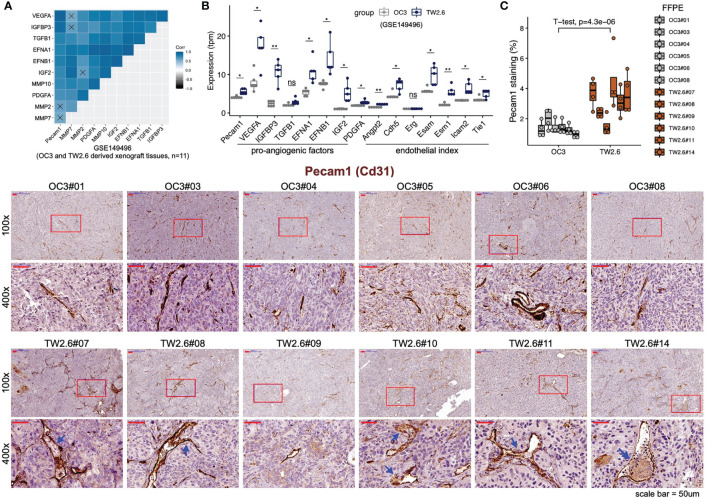
Statistical enrichment of microvessel density in the p-EMT TW2.6 tumor stroma. **(A)** Correlational expression matrix of stromal *Pecam1* and indicated proangiogenic genes in graft tumors (OC3, n=6; TW2.6, n=5). Crosses (×) indicate p > 0.05. **(B)** Box plots showing the expression of *Pecam1*, proangiogenic, and endothelial index (41) genes in xenograft tissues. Two-sample t-tests of means were used to evaluate significant differences. *p < 0.05, **p < 0.01, ns, not significant. **(C)** Quantitation (mean ± SEM of four 100x magnification fields for each section) and representative images of the indicated tissue sections stained for murine Pecam1 (brown). The p value of two-sample t-test of means is denoted. Blue arrowheads denote angiolymphatic invasions (ALIs) only detected in the TW2.6 tumors, a phenomenon consistent with our prior study (10). Note that TW2.6-NOG11 was not included in RNA-seq analysis.

To validate that the p-EMT TW2.6 stroma had higher angiogenesis processes than the OC3 group, we first performed correlational analysis of stromal *Pecam1* expression, a microvessel density surrogate, with that of each well-established proangiogenic and endothelial index gene recently established in > 10,000 human tumors (41). The results indicated that the expression of tumor cell-derived *VEGFA*, *IGFBP3*, *EFNA1*, *EFNB1*, *IGF2*, *PDGFA* and stroma-derived *Angpt2*, *Cdh5*, *Esam*, *Esm1*, *Icam2*, and *Tie1* was statistically correlated with that of *Pecam1* (**Figure 2A**) and elevated in p-EMT TW2.6 tumors (**Figure 2B**). In parallel, immunohistochemistry using a *Pecam1* antibody not only verified significantly increased vascular densities but also prominent angiolymphatic invasions in the p-EMT TW2.6 tissues compared to their mesenchymal OC3 counterparts (**Figure 2C**). Taken together, these results strongly suggest that a higher blood supply from the tumor stroma might contribute to fostering better growth of p-EMT TW2.6 cells *in vivo*.

### Tumor Cell-Excluded Macrophages Were Detected in p-EMT TW2.6 Tumors

The other prominent expression feature enriched in the TW2.6 stroma is immune-related molecular signatures, including various cytokines and chemokines (e.g., *Il6*, *Cxcl9*, *Cxcl10*, *Cxcl12*). At first, this seemed perplexing since prior results showed that stronger innate immunity was present in OC3 tumor cells (11). To resolve this confusion, we performed *in silico* enumeration of cell fractions available at the CIBERSORTx portal (27). We first employed ‘scRNA-seq HNSCC’ as a signature matrix, since both OC3 and TW2.6 cells are derived from the oral cavity. As expected, while no significant difference was detected in the tumor compartment (primarily consisting of ‘malignant’ cells), the ‘fibroblast’ cell fraction was significantly higher in the OC3 stroma than in the TW2.6 stroma, which is consistent with our prior results (11) (**Supplementary Figures 2A, B**). Importantly, the constituents of other immune cell populations did not differ significantly between the two groups. Alternatively, using LM22, the default signature matrix comprising cell type-specific genes derived from 22 leukocytes revealed two immune populations, i.e., ‘Macrophage-M1’ and ‘Tγδ‘, were statistically elevated in the TW2.6 stroma. Further delineation of hallmark genes comprising each LM22 cell type revealed that instead of authentic immune cell markers, increased expression of stromal *Cxcl9*, *Cxcl10*, *Cxcl11* (Macrophage-M1) and *Ccl5* (Tγδ) were likely to be the leading genes contributing to the statistical enrichments (**Supplementary Figures 2C, D**).

**Figure 3:**
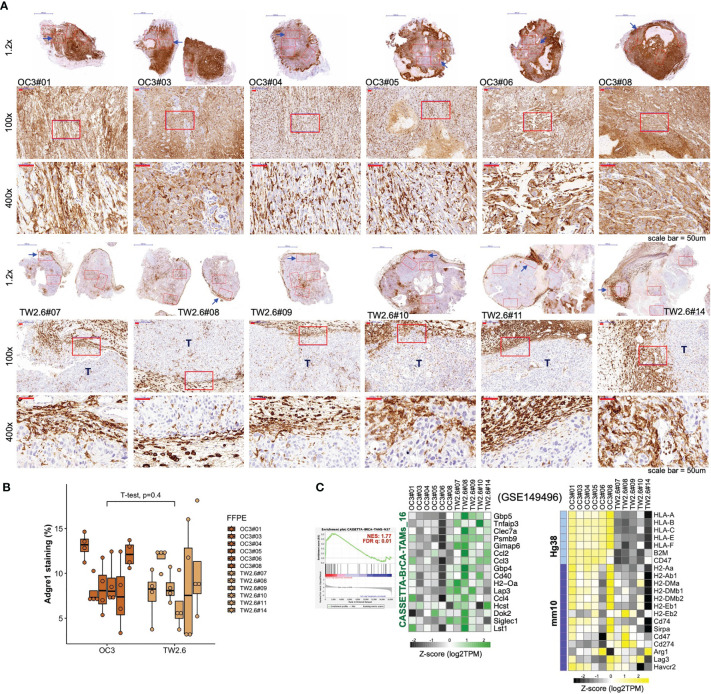
Macrophages in the OC3 and TW2.6 stroma are geographically different. **(A)** Representative images and **(B)** quantitation (mean ± SEM of four 100x magnification fields from each section) of the indicated tissue sections stained for murine Adgre1 (brown). The p value of two-sample t-test of means is denoted. **(C)** (Left) GSEA enrichment end-plot and heatmap showing signature genes of tumor-associated macrophages (43) that are statistically enriched in the TW2.6 stroma. (Right) Heatmap showing the expression of human major histocompatibility (MHC) class-I (Hg38)- and mouse MHC class-II (mm10)-related genes in each xenograft tissue.

Given that ‘macrophage’ represents the major (57.2 ± 6.7%) immune cell type residing in the host stroma by CIBERSORTx analysis, immunohistochemistry using an antibody against the panmacrophage marker Adgre1 (F4/80) was performed to visualize the infiltration of macrophages in each tumor section. Unexpectedly, the spatial locations of macrophages were significantly different between the OC3 and TW2.6 groups. In the majority of OC3 tissues, the tumor cells are interdigitated with irregular Adgre1-positive macrophages and are accompanied by high staining backgrounds. By contrast, in the TW2.6 group, Adgre1-positive prototypical macrophages were frequently detected in the tumor margins, a phenomenon reminiscent of ‘immune privileged’ sites detected in clinical head and neck cancer tissues (42). Note that no significant difference in macrophage staining was noticed between the OC3 and TW2.6 groups after background subtractions (**Figures 3A, B**), which is consistent with the CIBERSORTx results. Intriguingly, as depicted in **Figure 3C**, the simultaneous upregulation of murine signature genes of tumor-associated macrophages (43) and downregulation of major histocompatibility class II molecules required for antigen presentation in TW2.6 tissues reinforces the accumulation of anergic tumor-associated macrophages surrounding p-EMT TW2.6 tumor cells.

### OC3 and TW2.6 Tumors Are Enriched With Gene Signatures of Myofibroblastic and Inflammatory CAFs, Respectively

Recent scRNA-seq studies have shown that the inflammatory subtype of CAFs (iCAFs) secretes a variety of inflammatory mediators (e.g., IL6 and CXCL12), whereas the myofibroblastic counterpart (myCAFs) primarily expresses ECM molecules (e.g., FN1 and type I collagen) (18–23). Of special interest, by microarray-based transcriptomics, Costea et al. identified two distinct subtypes of CAFs from clinical oral cancer tissues, designated CAF-N (normal-like) and CAF-D (divergent) (44). CAF-N is intrinsically motile, secretory, proangiogenic, and hyaluronan-rich. CAF-D is less migratory and secretes high levels of TGFβ but is unresponsive to it. Compared to CAF-D, the CAF-N population displayed a greater (85.33% *vs*. 50%) and faster (7 *vs*. 14 days) tumor-promoting incidence of otherwise nontumorigenic oral dysplastic cells in the immunodeficient mice (NSG) and significantly deeper invasion of malignant cells in the 3D biometrices constructed *in vitro*. The authors proposed that a switch from an earlier secretory CAF-N to a later TGFβ^high^ CAF-D occurred during oral cancer progression.

**Figure 4:**
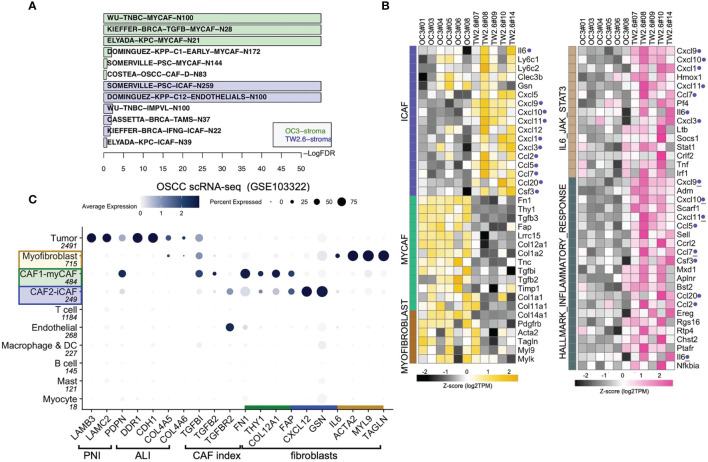
Molecular signatures of myCAF and iCAF were enriched in OC3 and TW2.6 stroma, respectively. **(A)** Bar chart showing cell types with significant enrichments (FDR < 5%) in OC3 or TW2.6 stroma by gene set enrichment analysis (GSEA). Cell type-specific gene matrices in each study (summarized in **Table 1**) were extracted and converted to gene matrix transposed (gmt) files used in GSEA. N, gene number of indicated gene matrix. **(B)** Heatmaps showing the expression of the indicated genes classified as stromal fibroblasts (left) or immune-related (right) molecular signatures. Blue dots refer to replicates present in both heatmaps. **(C)** Dot plot denotes RNA expression of the indicated genes (*x*-axis) across eleven cell types (*y*-axis) composing the scRNA-seq dataset. Dot size indicates the proportion of cells within the indicated cell type expressing the indicated gene; color intensity represents the binned count-based expression level [log(scaled normalized count + 1)] among expressing cells. OSCC, oral squamous cell carcinoma; PNI, perineural invasion; ALI, angiolymphatic invasion. CAF index is referred to (11).

To test the hypothesis that inflammatory cytokines detected in the TW2.6 stroma might come from the iCAF cell population, in GSEA we applied customized gene matrix transposed (gmt) file of each newly identified stromal cell population as summarized in **Table 1**. Interestingly, this approach explicitly assigned enrichment of myCAF and CAF-D to the OC3 stroma and iCAF, tumor-associated macrophages, endothelial cells, and perivascular-like cells to the TW2.6 stroma (**Figure 4A**). In addition, a great number of inflammatory mediators are overlapping genes of the iCAF and immune-related pathways (e.g., IL6/JAK/STAT3 and inflammatory responses), which supports our hypothesis that CAFs of the TW2.6 stroma might be the principle source for the immune-related signatures (**Figure 4B**).

**Table 1:** Gene matrices of stromal cell populations used in GSEA analysis.

yyyy/mm	Authors	Cancer	Stromal sub-populations
2013/07	COSTEA et al. (44)	OSCC	CAF-D, CAF-N
2019/04	CASSETTA et al. (43)	BRCA	TAM
2019/08	ELYADA et al. (18)	PDAC	myCAF, iCAF, apCAF
2020/02	DOMINGUEZ et al. (19)	PDAC	myCAF, iCAF, endothelials
2020/04	SOMERVILLE et al. (20)	PSC	myCAF, iCAF
2020/08	WU et al. (22)	TNBC	myCAF, iCAF, imPVL, dPVL
2020/09	KIEFFER et al. (21)	BRCA	myCAF, iCAF

OSCC, oral squamous cell carcinoma; BRCA, breast cancer; PDAC, pancreatic ductal adenocarcinoma; PSC, pancreatic stellate cell; TNBC, triple-negative breast cancer; CAF, cancer-associated fibroblast; TAM, tumor-associated macrophage; PVL, perivascular-like cell.

We were aware that two caveats are inherent to the current study: (1) immunodeficient NOG xenografts were used, which retained only limited innate immunity, and (2) human orthologous genes might not be entirely functional in mice. With these concerns, we performed cell type-gene expression analysis using the scRNA-seq dataset of oral cancer tissues in which the presence of CAF subtypes, including myCAFs and iCAFs, has been previously noted (8, 19, 21, 23). The results showed that except for *IL6*, which was enriched in the iCAF population of xenograft tissues but myofibroblasts in the clinical samples, hallmark genes for myCAFs, iCAFs, and myofibroblasts were fairly consistent between the xenograft and clinical oral cancer tissues, suggesting a certain extent of conservation in non-immune stromal cell populations preserved in the current study (**Figure 4C**).

### Statistically Higher Hyaluronan Accumulation Was Detected in p-EMT TW2.6 Tumors

Another notable feature repeatedly linked to the secretory iCAF population is increased levels of hyaluronan synthases, including *HAS1* and *HAS2* (18, 19, 44). Interestingly, in our system, significantly increased expression of synthases (*HAS2*, *HAS3*) and hyaluronidases (*HYAL1*, *HYAL3*, *HYAL4*) was detected in the tumor cells but not in the stromal portion of TW2.6 tumors (**Supplementary Figure 3A**). Subsequent experiments using Alcian blue staining of OC3 and TW2.6 xenograft tissues confirmed that prominent hyaluronan staining was detected in five out of six TW2.6 tissues compared to sporadic Alcian blue-positive mast cells revealed in the OC3 tissues (**Supplementary Figure 3B**). Thus, our data partly support prior scRNA-seq studies in that iCAFs are frequently present in a hyaluronan-rich tumor microenvironment.

### Translation of Xenograft Results Into Clinical Application

Thus far, our results provide evidence that certain stromal cell populations are preferentially associated with the most invasive yet uncommon EMT (OC3) or p-EMT (TW2.6) tumor cells, which is very different from a real clinical specimen that comprises cells at variable EMT states, e.g., only tumor cells at the invasion front display a p-EMT phenotype (6). A complementary experiment is to correlate stromal genes of interest with transcriptomic datasets from clinical bulk tissues, an approach we employed to identify the CAF index (summed expression of *TGFBI*, *TGFB2*, *TGFBR2*, *FN1*) (11). Specifically, the Puram oral cancer scRNA-seq (GSE103322) and NCKU-OrCA-40TN (GSE37991) datasets were chosen for cell type mapping and survival analysis, respectively. Of note, the latter is a microarray dataset comprising 40 matched pairs of betel quid-associated oral squamous cell carcinoma and adjacent normal tissues. Due to treatment-related death, only 38 cases were included for survival analysis. In addition, except for recurrence, none of the other clinical features were statistical covariates for overall survival in univariate Cox proportional hazards model assessment (**Table 2**), an inherent limitation of a small cohort. Further TCGA collections with larger sample sizes will be included as validation datasets to complement this caveat.

**Table 2:** Cox proportional hazards analysis of NCKU-OrCA-40TN.

Feature/Gene	HR (95% CI) p value^#^	Notes
Age	1.5 (0.55-4.2) p=0.42	above 49 (n=20) *vs*. under 49 (n=18)
Alcohol	1.3 (0.36-4.6) p=0.7	no (n=8) *vs*. yes (n=30)
Diagnosis	1.8 (0.58-5.8) p=0.3	buccal (n=14) *vs*. tongue (n=24) Carcinoma
ALI	1.9 (0.67-5.3) p=0.23	no (n=20) *vs*. yes (n=18)
PNI	2.7 (0.76-9.6) p=0.12	no (n=14) *vs*. yes (n=24)
LNmeta	1.8 (0.65-5) p=0.26	no (n=21) *vs*. yes (n=17)
TNMstage	1.8 (0.58-5.8) p=0.3	I/II (n=14) *vs*. III/IVA (n=24)
TxModality	1 (0.36-2.8) p=0.99	Op only (15) *vs*. OpRT or OpCCRT (17 + 6)
Recurrence	7 (2.5-20) p=0.00024***	no (n=28) *vs*. yes (n=10)
EMT.Salt	4.2 (1.3-14) p=0.015*	higher score toward mesenchymal, composed of 14 genes
EMT.76GS	0.16 (0.043-0.56) p=0.0044**	higher score toward epithelial, composed of 76 genes
CAF index	13 (2.8-57) p=0.0011**	TGFBI+TGFB2+TGFBR2+FN1
TGFBI	11 (2.4-50) p=0.0021**	CAF index, myCAF
TGFB2	4.9 (1.4-17) p=0.015*	CAF index, myCAF
TGFBR2	1.3 (0.49-3.7) p=0.57	CAF index, endothelial cell
FN1	1.8 (0.63-5) p=0.28	CAF index, myCAF
THY1	1.1 (0.38-2.9) p=0.92	myCAF
COL12A1	3.2 (1.1-9.5) p=0.041*	myCAF
FAP	2.6 (0.88-7.6) p=0.085	myCAF, iCAF
CXCL12	1 (0.37-2.8) p=0.98	iCAF
GSN	0.55 (0.19-1.6) p=0.27	iCAF
IL6	3.4 (1.1-11) p=0.038*	myofibroblast, iCAF
ACTA2	0.6 (0.21-1.7) p=0.34	myofilbroblast
MYL9	1.1 (0.38-2.9) p=0.91	myofilbroblast
TAGLN	1.4 (0.49-3.8) p=0.55	myofilbroblast
HAS1	1.3 (0.45-3.5) p=0.66	hyaluronic acid synthase, myCAF
HAS2	5.8 (1.6-21) p=0.0068**	hyaluronic acid synthase, myCAF
HAS3	0.42 (0.14-1.2) p=0.11	hyaluronic acid synthase, tumor cell
HYAL1	0.15 (0.042-0.55) p=0.0039**	hyaluronidase, endothelial cell
HYAL2	1 (0.36-2.8) p=1	hyaluronidase, endothelial cell
HYAL3	0.78 (0.28-2.2) p=0.63	hyaluronidase, mast cell
HYAL4	0.85 (0.31-2.3) p=0.75	hyaluronidase, T cell
PECAM1	1.7 (0.6-4.7) p=0.32	microvessel density surrogate, endothelial cell

^#^Significance codes: ***< 0.001, **< 0.01, *< 0.05. ALI, angiolymphatic invasion; PNI, perineural invasion; LNmeta, lymph node metastasis; Op, operation; OpRT, operation plus radiotherapy; OpCCRT, operation plus concurrent chemoradiotherapy.

**Figure 5:**
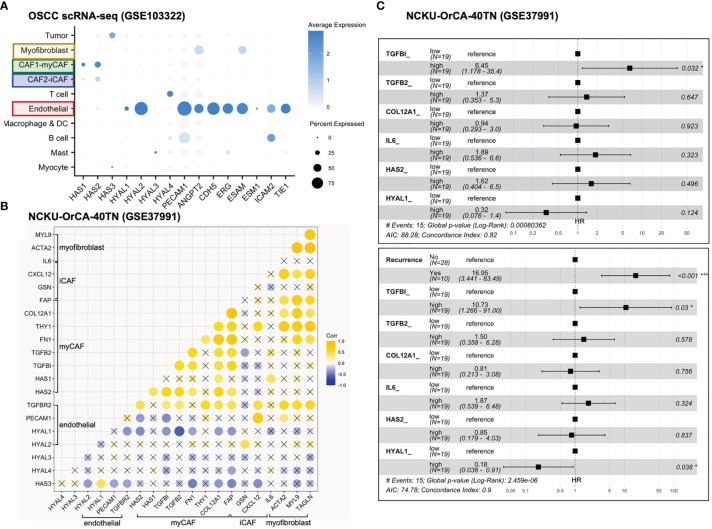
Stromal *TGFBI* and *HYAL1* are poor and protective prognostic biomarkers, respectively, for NCKU-OrCA-40TN. **(A)** Dot plot denotes the cell origins of hyaluronan synthase (HAS1–3), hyaluronidase (HYAL1–4) and endothelial hallmark genes (PECAM1 ~ TIE1), which were included as controls. Dot size indicates the proportion of cells within the indicated cell type expressing the indicated gene; color intensity represents the binned count-based expression level [log(scaled normalized count + 1)] among expressing cells. OSCC, oral squamous cell carcinoma. **(B)** Correlational expression matrix of stroma-originating genes in the NCKU-OrCA-40TN cohort. Crosses (×) indicate p > 0.05 **(C)** Forest plots representing the prognostic hazard ratios (HR) of overall survival assessed by 6 stromal genes (upper plot) or stromal genes plus cancer recurrence (lower plot). *p < 0.05, ***p < 0.001.

First, genes identified in the xenograft experiments were validated for their primary origins of expression by cell typing of the scRNA-seq database, including myofibroblasts (*IL6*, *ACTA2*, *MYL9*, *TAGLN*), myCAFs (*TGFBI*, *TGFB2*, *FN1*, *THY1*, *COL12A1*, *FAP*, *HAS1*, *HAS2*), iCAFs (*FAP*, *CXCL12*, *GSN*), endothelial cells (*PECAM1, TGFBR2*, *HYAL1*, *HYAL2*), mast cells (*HYAL3*), T cells (*HYAL4*) and tumor cells (*HAS3*) (**Figures 4C**, **5A**). Next, in the 40 tumor tissues of NCKU-OrCA-40TN, the *Pearson* correlation coefficient of each paired gene was computed and clustered by cell type to inspect their expression consistency. As depicted in **Figure 5B**, (1) the expression of myCAF genes correlated with each other the best. (2) Myofibroblast genes share partial similarities to iCAFs and myCAFs, suggesting myofibroblasts might be progenitors of both CAF subtypes. (3) The expression of *PECAM1*, a microvessel density proxy, was inversely correlated with myCAF-*HAS2* and *TGFBI* but significantly correlated with iCAF-*CXCL12* and myofibroblast-*ACTA2*. (4) The expression of *HYAL1* is inversely related to myCAF genes but positively related to tumor cell *HAS3*.

Third, a univariate Cox proportional hazards model was performed to compute the hazard ratios (HRs) of overall survival associated with each stroma-originating gene in the NCKU-OrCA-40TN cohort. Accordingly, 6 out of 20 selected genes revealed statistical significance, including *TGFBI* (HR 11, 95% CI 2.4–50, p=0.0021*)*, *TGFB2*, *COL12A1*, *IL6, HAS2*, and *HYAL1* (HR 0.15, 95% CI 0.042–0.55, *p=*0.0039) (**Table 2**). Multivariable Cox analysis revealed these 6 genes are confounding covariates (**Figure 5C** upper panel), which was not unexpected since 4 of them (*TGFBI*, *TGFB2*, *COL12A1* and *HAS2*) are derived from myCAFs. As cancer recurrence is a known prognostic factor for overall survival, we included it in multivariate Cox analysis. The results showed that *TGFBI* and *HYAL1*, respectively, remained statistically significant in multivariate Cox models (**Figure 5C** lower panel). These data suggested that both genes could serve as robust prognostic factors in betel quid-oral cancer. It is worth noting that *TGFBI* and *HYAL1* should act independently rather than combined, as both lost partial significance in bivariate Cox assessment (*TGFBI* 8.23 95% CI 1.66–40.79, p=0.01, *HYAL* 0.23, 95% CI 0.06–0.88, p=0.032).

**Figure 6:**
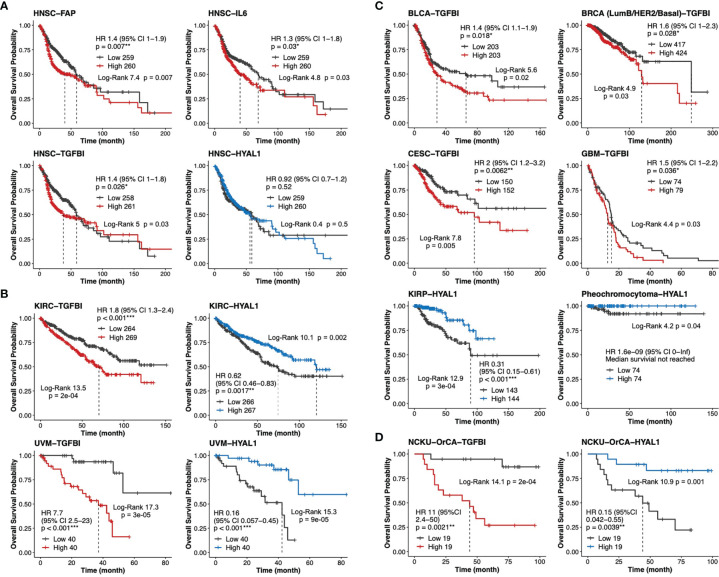
Validation of *TGFBI* and *HYAL1* in The Cancer Genome Atlas (TCGA) datasets. **(A)** Dichotomized Kaplan-Meier curves of estimated overall survival for *FAP*, *IL6*, *TGFBI* and *HYAL1* in the head neck cancer cohort (HNSC, n=519). **(B)** Kaplan-Meier curves for *TGFBI* and *HYAL1* in kidney renal clear cell carcinoma (KIRC, n=533) and uveal melanoma (UVM, n=80). **(C)** Kaplan-Meier curves for *TGFBI* in bladder urothelial carcinoma (BLCA, n=406), invasive breast cancer (BRCA, n=841), cervical squamous cell carcinoma and endocervical adenocarcinoma (CESC, n=302), and glioblastoma multiforme (GBM, n=153); *HYAL1* in kidney renal papillary cell carcinoma (KIRP, n=287), and pheochromocytoma (n=148). **(D)** Kaplan-Meier curves for *TGFBI* and *HYAL1* in the NCKU-OrCA-40TN cohort (GSE37991, n=38). *p < 0.05, **p < 0.01, ***p < 0.001.

To validate our findings in larger TCGA cancer datasets, we first performed univariable Cox analysis and Kaplan–Meier curves stratified by low- and high-expression groups for each of the 20 stromal genes in the head and neck cancer cohort (HNSC, n=519). The results indicated that while high expression levels of *TGFBI*, *FAP* and *IL6* were statistically prognostic for poorer survival; the expression of *HYAL1* did not reach statistical significance for better survival (**Figure 6A**). It is worth noting that HNSC comprises 11 anatomic subsites including tonsil and larynx, such heterogeneity in tissue source might interfere with precisely identifying stromal biomarkers specific to each HNSC subtype. Next, same approach was used to evaluate *TGFBI* and *HYALI* in the other 32 TCGA cancer types. Interestingly, kidney renal clear cell carcinoma (KIRC, n=533) and uveal melanoma (UVM, n=80) concurrently displayed *TGFBI* and *HYAL1* as poor and good biomarkers, respectively (**Figure 6B**). In addition, the expression of *TGFBI* was also a poor indicator for bladder urothelial carcinoma (BLCA, n=406), breast invasive carcinoma (BRCA, n=841), cervical squamous cell carcinoma and endocervical adenocarcinoma (CESC, n=302), and glioblastoma multiforme (GBM, n=153); and the expression of *HYAL1* was a statistical protective marker for kidney renal papillary cell carcinoma (KIRP, n=287) and pheochromocytoma (n=148) (**Figure 6C**). Taken together, the prognostic values of *TGFBI* and *HYAL1* identified from betel quid-associated oral cancer tissues (**Figure 6D**) were recapitulated by seven (n=2834) and four (n=1048) TCGA cancer types, respectively.

## Discussion

Through meta-analysis of disease-matching transcriptomic xenograft tumor tissue and scRNA-seq datasets, this study extracted 20 stromal genes representative of myCAFs, iCAFs, myofibroblasts, and endothelial cells in a betel quid-oral cancer cohort comprising 40 tissues (**Table 2**). In univariate Cox proportional hazards assessment of overall survival, 6 out of these 20 genes exhibited statistical predictability. In multivariate Cox analysis interacting with cancer recurrence, *TGFBI* and *HYAL1* remained statistically significant for poor and good prognosis, respectively (**Figure 5C**). In thirty-three TCGA transcriptomic cohorts, *TGFBI* was a poor indicator of seven cancer types, including head and neck cancer; *HYAL1* was a protective marker for four cancer types, including kidney renal clear cell carcinoma and uveal melanoma.

With an unprecedented pace, scRNA-seq methodology has successfully deciphered the complexity and heterogeneity of tumor ecosystems composed of tumor cells and various stromal components. Among these, the two distinct molecular subtypes of cancer-associated fibroblasts, iCAFs and myCAFs, attracted special attention due to their relevance to cancer cell invasion and treatment resistance. In oral cancer, malignant p-EMT cells located at the invasion front are in proximity to CAFs (FAP+PDPN+) and are statistically associated with nodal metastasis and perineural invasion (8, 9). In breast cancer, while iCAFs (PDGFRB+, ACTA2-, CD34+, MCAM-) were implicated in cytotoxic T cell dysfunction of tripe-negative breast cancer (22), myCAFs (ecm) and myCAFs (TGFβ) were shown to be the primary resistance elements of immunotherapies (21). In pancreatic ductal adenocarcinoma, increased levels of the myCAF (LRRC15+) signature correlated with poor response to anti-PD-L1 therapy in an immunotherapy clinical trial (19). In a murine melanoma model, iCAFs (S1 immune), myCAFs (S2 desmoplastic), and myofibroblasts (S3 contractile, ACTA2^high^) were temporally linked to disease progression (23).

Another stromal population, vascular endothelial cells, is also therapy relevant. In a comprehensive transcriptomic study comprising 10,767 human tumors with variable extents of vascularity, Kahn et al. revealed that both the endothelial index (**Figure 2B**) and vascular microenvironment signatures are independent predictors of disease outcome (41). In this regard, motile and secretory iCAFs (and their equivalents with different designations) were linked to angiogenesis, variably implicated by the increased expression of *CXCL12/SDF-1*, *VEGFA*, *CCL2*, *FGF*s, *PDGF*s, and hyaluronic acid synthase (*HAS1*, *HAS2*) (18, 19, 23, 44). Distinct from the other proangiogenic factors, hyaluronan is a linear, anionic polysaccharide required for normal tissue homeostasis. The degradation of high molecular weight (HMW-HA, > 500 kDa) to low molecular weight hyaluronan (LMW-HA, 7–200 kDa) is mediated by hyaluronidases (HYAL1–4). Emerging evidence indicates that while LMW-HA participates in neoangiogenesis, tumor cell proliferation, migration, and invasion; in an established tumor, accumulated HMW-HA increases the intratumor interstitial fluid pressure, which blocks neoangiogenesis at the cost of reducing immune surveillance and drug delivery efficacy [reviewed in 45)].

In the oral cancer scRNA-seq study, the expression of *TGFBI* came from myCAFs, myofibroblasts and tumor cells (**Figure 4C**). TGFBI is a secretory extracellular matrix protein that mediates binding to other matrices, including fibronectin, laminin, and collagen of types I, II, IV, etc. As a direct target of TGFβ, TGFBI plays a tumor-suppressive role in early precancerous lesions but acts to promote tumor progression in later stages. Indeed, TGFBI is one of the p-EMT hallmark proteins present in oral cancer tissues (8) and is implicated in DDR1-mediated angiolymphatic invasions in the NCKU-OrCA-40TN cohort (10). Whether TGFBI produced by myCAFs and myofibroblasts is functionally different from that secreted by tumor cells awaits further investigation.

As the pathologic roles of otherwise nonmalignant stromal cell populations are beginning to emerge, prognostic biomarkers originating from the tumor stroma might illuminate a new avenue for cancer control. Indeed, while we should be more cautious about defining the tumor cell EMT spectrum in a real specimen (6), it is inspiring to learn that “the CAF-targeted therapy will take its place in the toolkit of the oncologist within the next 10 years” (46)!

## Conclusions

Through integrative studies of disease-matching xenograft tumor and scRNA-seq datasets, we established nonmalignant stromal cell populations preferentially cohabitate with oral cancer cells residing in EMT and p-EMT states, the most invasive and deleterious components within a tumor. Hallmark genes representative of myofibroblasts, myCAFs, iCAFs, and endothelial cells were assessed for Cox hazard ratios and Kaplan-Meier curves of overall survival in clinical datasets. MyCAF-*TGFBI* and endothelial-*HYAL1* were poor and good prognosis markers, respectively, for 40 betel quid-associated oral cancer tissues. In 33 TCGA datasets, *TGFBI* was recapitulated as a poor indicator for seven cancer types, including head and neck cancer comprising 519 patients. Our results not only disclose novel targets for oral cancer control, but also provide feasible applications, e.g, a single immunohistochemical assay of TGFBI from treatment naïve or recurred tumor biopsies, to assist clinical decision-making.

## Data Availability Statement

The datasets presented in this study can be found in online repositories. The names of the repository/repositories and accession number(s) can be found below: GSE149496, GSE150469.

## Ethics Statement

The studies involving human participants were reviewed and approved by the Research Ethics Committee of National Health Research Institutes (Protocol No: EC1040407-E) for the use of clinical samples and patient data. Written informed consent for participation was not required for this study in accordance with the national legislation and the institutional requirements. The animal study was reviewed and approved by the Institutional Animal Care and Use Committee of National Health Research Institutes, Taiwan (Protocol No: NHRI-IACUC-106057-M1-A).

## Author Contributions

YHL, YFC, PRC, JWL, and SFL conceptualized this study. YCK, TYL, and YLC prepared xenograft tissues. YHL and YLC performed immunohistochemical assays and histological staining. YHL, TYL, YCK, and SFL performed bioinformatic analyses. JRH provides patient data. JRH, YFC, PRC, and JYC interpreted clinical data and survival analyses. SGS, JWL, JLY, and SFL participated in formal analysis, result validation, and are major contributors in writing the manuscript. All authors contributed to the article and approved the submitted version.

## Funding

This study was supported by the Taiwan National Health Research Institutes (NHRI CA-110-PP-05), Ministry of Health and Welfare (MOHW 110-TDU-B-212-144013 and -144026) and the Ministry of Science and Technology (MOST 108-2320-B-400-019). The NHRI institutional library paid for the open access publication fees for this manuscript.

## Conflict of Interest

The authors declare that the research was conducted in the absence of any commercial or financial relationships that could be construed as a potential conflict of interest.

## Publisher’s Note

All claims expressed in this article are solely those of the authors and do not necessarily represent those of their affiliated organizations, or those of the publisher, the editors and the reviewers. Any product that may be evaluated in this article, or claim that may be made by its manufacturer, is not guaranteed or endorsed by the publisher.
