# Supplementary material for: Identification of Prognostic Biomarkers Originating From the Tumor Stroma of Betel Quid-Associated Oral Cancer Tissues
Source: Front Oncol. 2021 Nov 16;11:769665. doi: 10.3389/fonc.2021.769665 (PMC8637169; doi:10.3389/fonc.2021.769665)
Supplement: Supplementary file 1 [file DataSheet_1.pdf]

frontiers

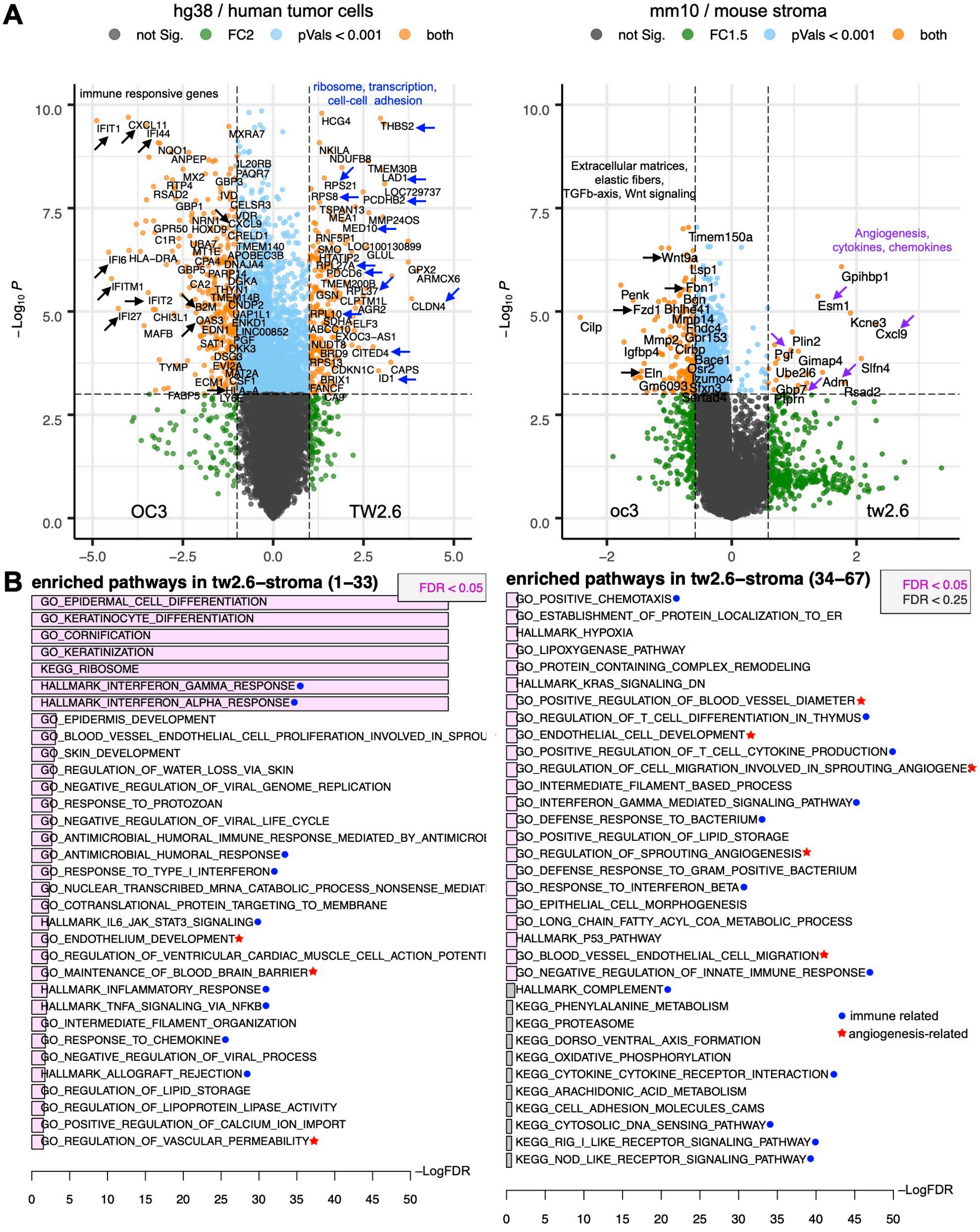

Supplementary Figure 1. Differentially expressed genes (DGEs) between the OC3 and the TW2.6 xenograft tissues. **(A)** Volcano plots showing DEGs in the tumor (left) and the stroma (right) compartments. In each plot, OC3 is on the left; TW2.6 is on the right. **(B)** Bar plots denote molecular processes/GO terms enriched in the TW2.6 stroma by gene set enrichment analysis.

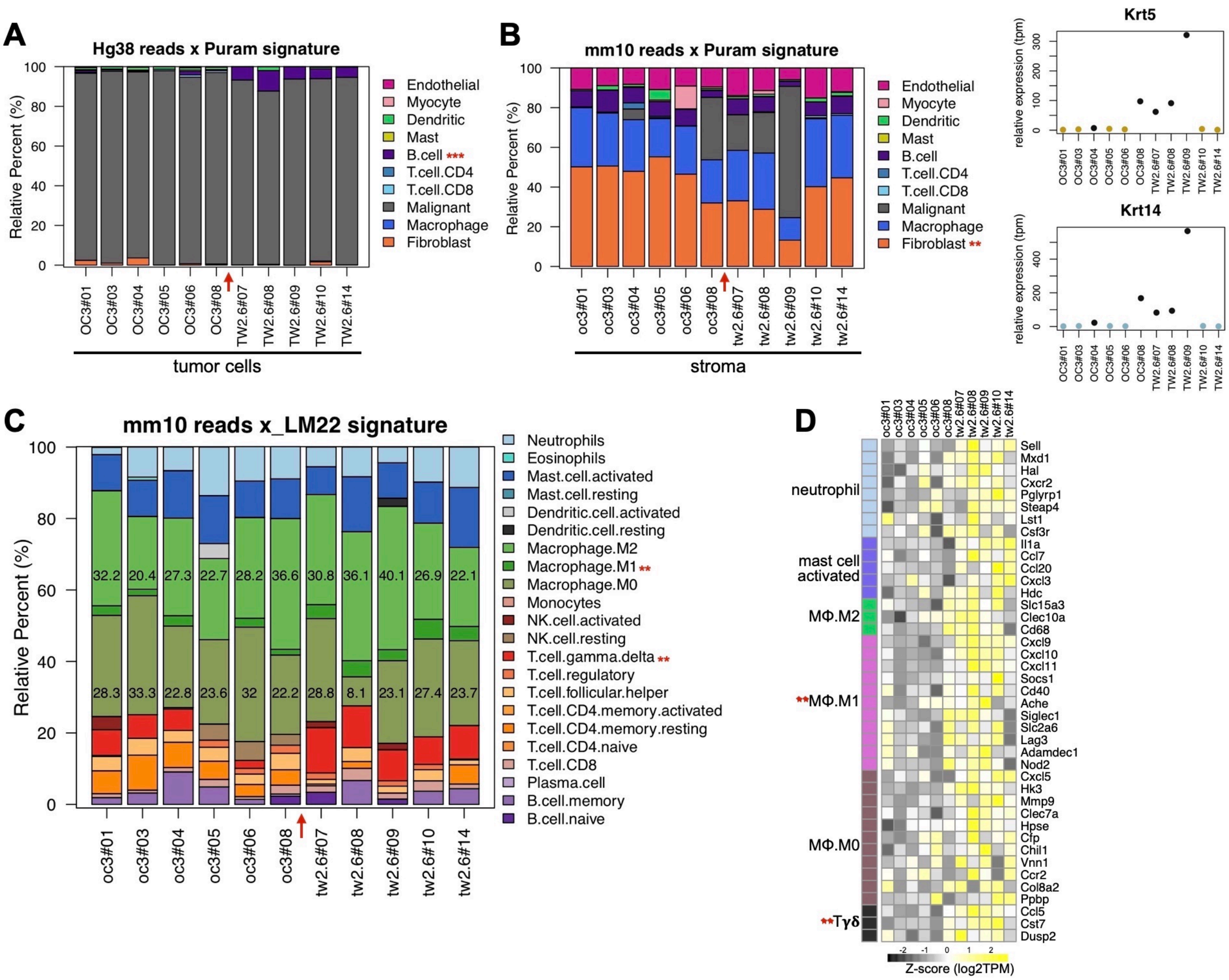

Supplementary Figure 2. *In silico* cellular fractionation of OC3 and TW2.6 xenograft tissues via CIBERSORTx. **(A-B)** The 'single cell RNA-seq HNSCC' was used as signature matrix. The expression matrices of tumor cells (Hg38 reads) and stroma components (mm10 reads) were enumerated. Dot pots showing expression of basal cytokeratin markers, *Krt5* and *Krt14*, are associated with the stromal 'Malignant' (dark-gray) fractions. **(C)** The 'LM22' was used as signature matrix; only the stromal compartments (mm10 reads) were enumerated. **(D)** Heatmap showing genes with significant enrichment scores of each indicated cell fractions. Two-sample t-test was used to evaluate statistical differences between OC3 and TW2.6. *p < 0.05, **p < 0.01, ***p < 0.001.

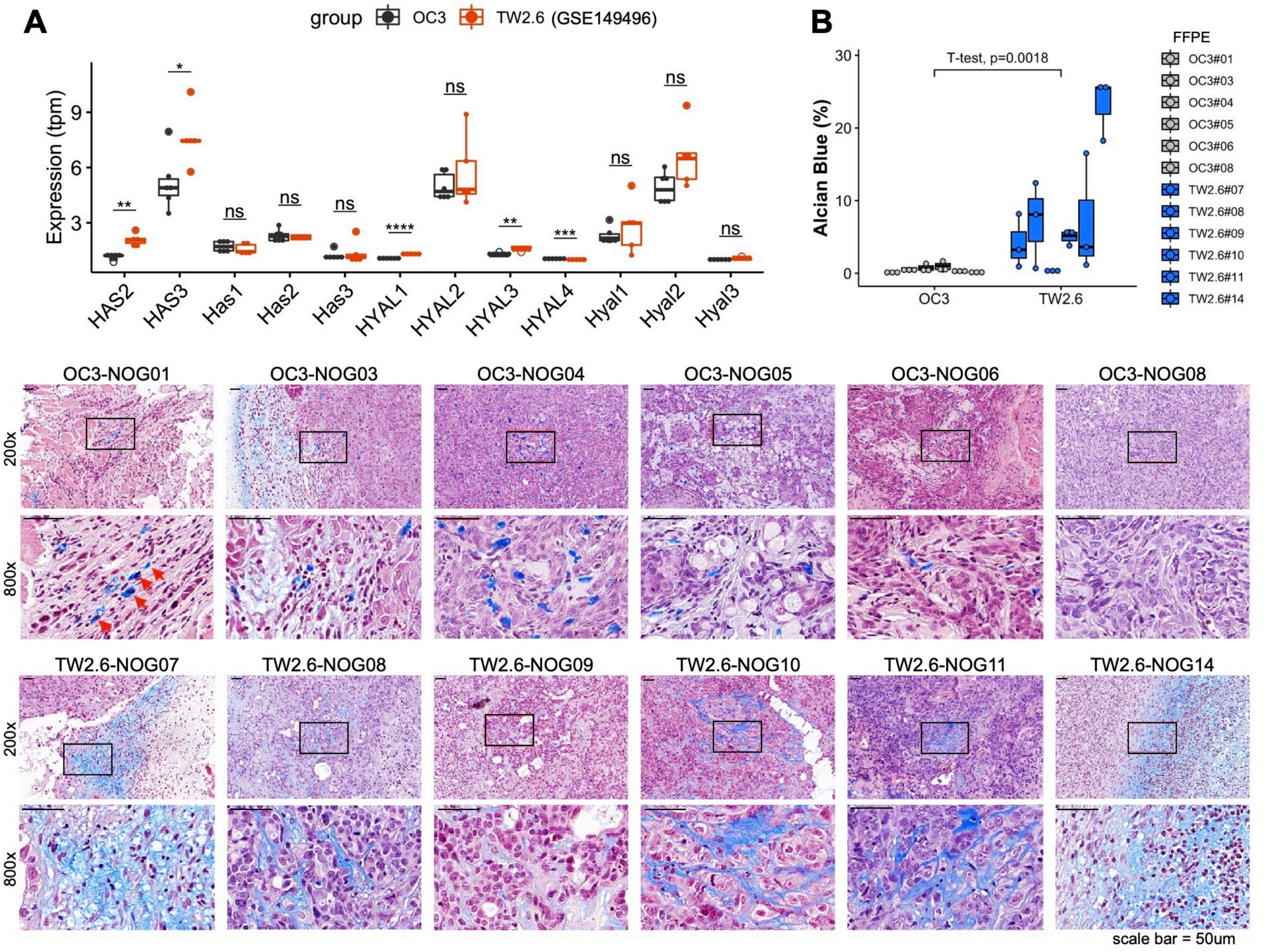

Supplementary Figure 3. Statistical enrichment of Alcian blue staining in the stroma of TW2.6 xenograft tissues. **(A)** Box plots showing the expression of hyaluronic acid synthase (*HAS2*, *HAS3*, *Has1*, *Has2*, *Has3*) and hyaluronidase (*HYAL1*, *HYAL2*, *HYAL3*, *HYAL4*, *Hyal1*, *Hyal2*, *Hyal3*) genes in the OC3 and TW2.6 xenograft tissues. *, $p < 0.05$; **, $p < 0.01$; ***, $p < 0.001$; ****, $p < 0.0001$; ns, not significant. **(B)** Quantitation (mean ± SEM of three 200x magnification fields from each section) and representative images of the indicated tissue sections stained for Alcian blue (light blue). The *p*-value of two-sample t-test of means is denoted. Red arrowheads in OC3-NOG01 (800x) indicate mast cells with Alcian blue staining.
